# Management of Facial Immune Checkpoint Inhibitor-Induced Vitiligo with Topical Ruxolitinib: Quantitative Assessment Using a Semi-Automatic Tool

**DOI:** 10.3390/curroncol33050300

**Published:** 2026-05-21

**Authors:** Thomas Breakell, Paolo Neri, Léonie A. N. Staats, Rafaela Kramer, Carola Berking, Michael Erdmann, Anke Hartmann

**Affiliations:** 1 Department of Dermatology, Uniklinikum Erlangen, Deutsches Zentrum Immuntherapie (DZI), Friedrich-Alexander-Universität Erlangen-Nürnberg, 91054 Erlangen, Germany; leonie.staats@uk-erlangen.de (L.A.N.S.); rafaela.kramer@uk-erlangen.de (R.K.); carola.berking@uk-erlangen.de (C.B.); michael.erdmann@uk-erlangen.de (M.E.); anke.hartmann@uk-erlangen.de (A.H.); 2 Comprehensive Cancer Center Erlangen-EMN and CCC Alliance WERA, 91054 Erlangen, Germany; 3 Bayerisches Zentrum für Krebsforschung (BZKF), 91052 Erlangen, Germany; 4 Department of Civil and Industrial Engineering, University of Pisa, 56122 Pisa, Italy; paolo.neri@unipi.it

**Keywords:** immune checkpoint inhibitor, immune-related adverse event, vitiligo, ruxolitinib, JAK inhibitor, melanoma

## Abstract

Immune checkpoint inhibitors (ICIs) have substantially improved outcomes in advanced melanoma but are frequently linked to immune-related adverse events (irAEs). Vitiligo is a common cutaneous irAE and has been consistently associated with improved patient outcome, including prolonged progression-free and overall survival. It also represents significant visual stigma, particularly when the face is involved. Traditional treatment comprises topical steroids, calcineurin inhibitors, laser, and phototherapy which often have insufficient effects. Since 2023, the first approved drug for non-segmental vitiligo (NSV) with facial involvement, the topical Janus kinase inhibitor ruxolitinib, has been available. However, experience with its use in ICI-induced vitiligo remains limited. In this exploratory analysis, three patients who developed facial vitiligo following ICI therapy applied 1.5% ruxolitinib cream to affected facial areas twice daily. After six (two patients), and twelve months (one patient), extensive repigmentation was observed, quantified at 95.7%, 78.9%, and 99.1% using a novel semi-automatic tool. Quality-of-life questionnaires showed mean reductions of 57.6% (Vitiligo DLQI) and 68.2% (Vitiligo-specific Quality of Life) in disease burden. Treatment was associated with substantial repigmentation without observed side effects. Further evaluation in larger, prospective cohorts is warranted to better define treatment effects, clinical applicability, and long-term safety.

## Simple Summary

Immunotherapy has greatly prolonged survival for patients with advanced melanoma but can cause multiple side effects, including vitiligo, a loss of skin pigmentation. Although the development of vitiligo is linked to better cancer outcomes, the visual patches can be distressing for patients and negatively affect quality of life. Treatment options are often unsatisfactory. In this report, we describe three patients who developed facial vitiligo during or after immunotherapy and were treated with a cream containing ruxolitinib, an approved medication for vitiligo. Skin repigmentation and changes in quality of life were assessed using clinical photographs and questionnaires. Treatment with ruxolitinib was associated with marked facial repigmentation and improvements in quality of life, without observed side effects. These findings suggest that ruxolitinib cream may represent a supportive-care option to enhance well-being in patients with melanoma affected by vitiligo caused by immunotherapy.

## 1. Introduction

Since the introduction of immune checkpoint inhibitors (ICIs) for metastatic and locally advanced melanoma, patient outcomes have vastly improved. ICI enhance the immune response against melanoma cells; however, they can also attack the body’s own tissue and cause a plethora of immune-related adverse events (irAEs) in virtually all organ systems by overactivating the immune system. Most commonly the skin, colon and endocrine systems are affected [1].

The incidence of cutaneous irAEs has been reported at 30 to 60%. Sorted from most to least frequent these include maculopapular rash, pruritus, eczematous eruptions, xerosis cutis, vitiligo, lichenoid and psoriasiform eruptions, bullous pemphigoid, and severe reactions including Stevens–Johnson syndrome and toxic epidermal necrolysis [2,3,4].

In patients with melanoma, the management of visible irAEs represents an important aspect of supportive oncologic care.

ICI-induced vitiligo has an incidence of approximately 6.6% and usually manifests within weeks to months of starting ICI therapy [5].

Its pathogenesis resembles, clinically as well as in pathogenesis, non-segmental vitiligo (NSV), formerly known as vitiligo vulgaris, a common autoimmune disorder. Both are caused by the selective destruction of endogenous melanocytes, and as these share antigens with melanoma cells, the ICI-activated immune system can confuse the two and cause depigmentation [6,7].

Strikingly, ICI-induced vitiligo has been shown to be associated with improved response to therapy, increased progression-free survival and overall survival in patients with melanoma, making it a positive prognostic marker in several studies [8,9,10,11,12,13,14].

Traditionally, treatment for NSV includes topical steroids and calcineurin inhibitors, as well as laser and phototherapy, generally used as off-label therapies [15].

In 2023, a cream containing the Janus kinase (JAK)-inhibitor ruxolitinib was approved as the first topical medication for the treatment of NSV affecting the face. Ruxolitinib inhibits JAK1- and JAK2-mediated interferon-γ signalling, disrupting the JAK-STAT (Signal Transducer and Activator of Transcription)-driven inflammatory cascade that promotes T-cell-mediated destruction of melanocytes in vitiligo [16]. In the phase III trials TRuE-V1 and -V2, minimal side effects such as itching and acne at the application site were observed in 5% and 6% of cases, respectively, with no systemic side effects or statistically significant laboratory changes [17].

Based on shared immunopathological features between ICI-induced vitiligo and NSV, topical ruxolitinib was considered as a therapeutic option for ICI-induced vitiligo.

Since ruxolitinib is not significantly absorbed transdermally, a major advantage of local application is the low risk of systemic side effects and relevant interference with the underlying oncologic disease and its therapy.

At present, the extent and patient impact of ICI-induced vitiligo are described primarily in qualitative terms in routine clinical practice. Objective, quantitative approaches to assess facial involvement and to relate visible disease burden to patient-reported outcomes are insufficient, limiting comparability across reports.

Despite recognition of ICI-induced vitiligo as a distinct irAE, evidence regarding its therapeutic management is insufficient, with the available literature primarily comprising heterogeneous observational data and descriptive reports that, despite increasing cumulative patient numbers, remain limited in terms of standardised quantitative assessment and systematic treatment evaluation [18,19,20]. As a result, treatment strategies are largely extrapolated from NSV, and condition-specific evidence is lacking.

In this context, exploratory analyses may provide valuable preliminary insights, particularly when incorporating objective and reproducible outcome measures.

This manuscript therefore aims to explore the feasibility of a standardised quantitative approach combining semi-automatic image analysis with validated quality-of-life instruments to elucidate the effect of topical ruxolitinib on facial ICI-induced vitiligo in a real-world clinical setting.

## 2. Materials and Methods

We describe three patients with metastatic or locally advanced melanoma who received ICI therapy and developed vitiligo affecting their faces during or after treatment. All patients suffered from considerable psychological distress due to the visible facial depigmentation caused by ICI-induced vitiligo. Therefore, the face was the main target site of treatment, with the aim of improving the patients’ quality of life.

Patients were identified retrospectively at a single department of dermatology university clinic. All patients with facial ICI-induced vitiligo treated with topical ruxolitinib identifiable in the institutional clinical records were included in the analysis.

Ruxolitinib 1.5% cream (Opzelura^®^, Incyte Dermatology, Morges, Switzerland) was prescribed as part of routine clinical practice and applied twice daily in a thin layer to affected areas of the face on clean and dry skin.

Treatment decisions were made on clinical grounds with the aim of achieving repigmentation and improving associated quality of life. Follow up was six months (2 patients) and twelve months (1 patient), respectively.

The impact of ICI-induced vitiligo on the patients’ quality of life was assessed using the standardised Dermatology Life Quality Index questionnaire (DLQI) that measures the burden of skin diseases and allows assessment of effectiveness of interventions based on patient perspective, with four added questions for vitiligo (Vitiligo DLQI, vDLQI) (“How strongly did you have to protect your skin from the sunlight or have difficulty tolerating sunlight?”; “How worried were you that your skin changes might get worse?”; “How strongly did your skin condition affect your sleep?”; “How depressed or sad did your skin condition make you feel?”), with a minimum score of 0 and maximum score of 42 [21].

Additionally, the Vitiligo-specific Quality of Life (VitiQoL) score with questions more closely tailored to patients with vitiligo was employed, with a minimum score of 0 and maximum score of 90 [22].

**Figure 1:**
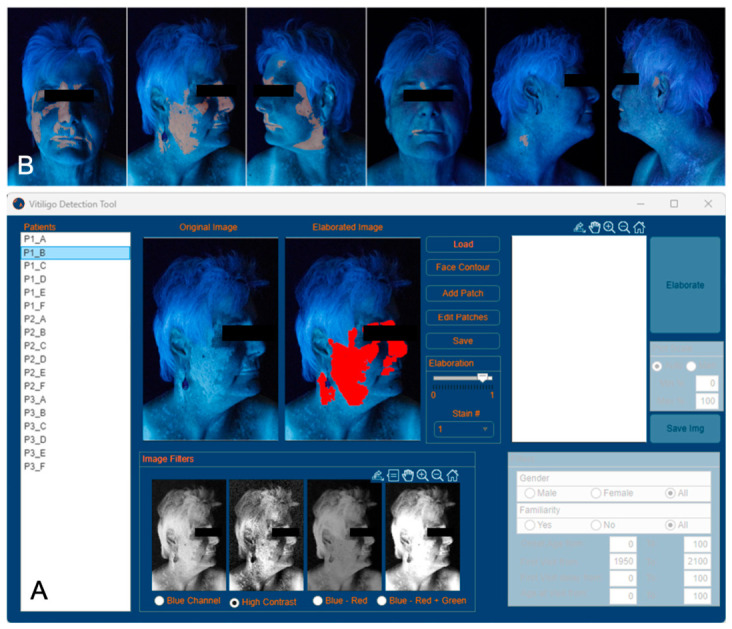
Graphical interface of the vitiligo detection tool. (**A**): Overview of the elaboration software, (**B**): results for Patient 1.

**Figure 2:**
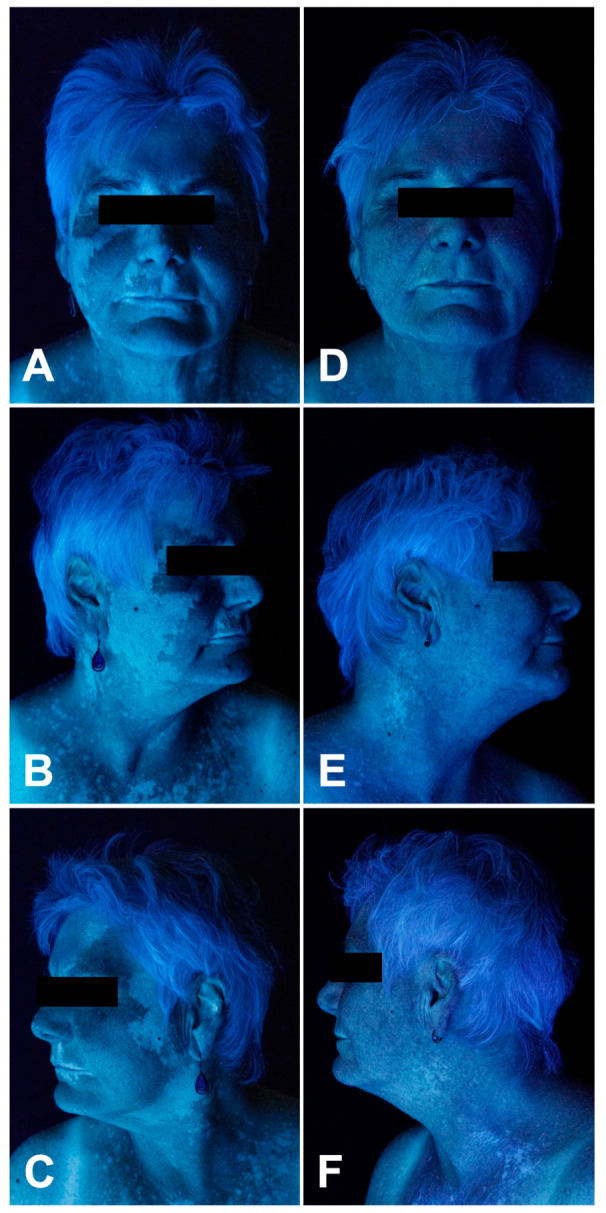
Patient 1: Clinical skin findings before (**A**–**C**) and after (**D**–**F**) six months of topical ruxolitinib 1.5% cream applied twice daily.

**Figure 3:**
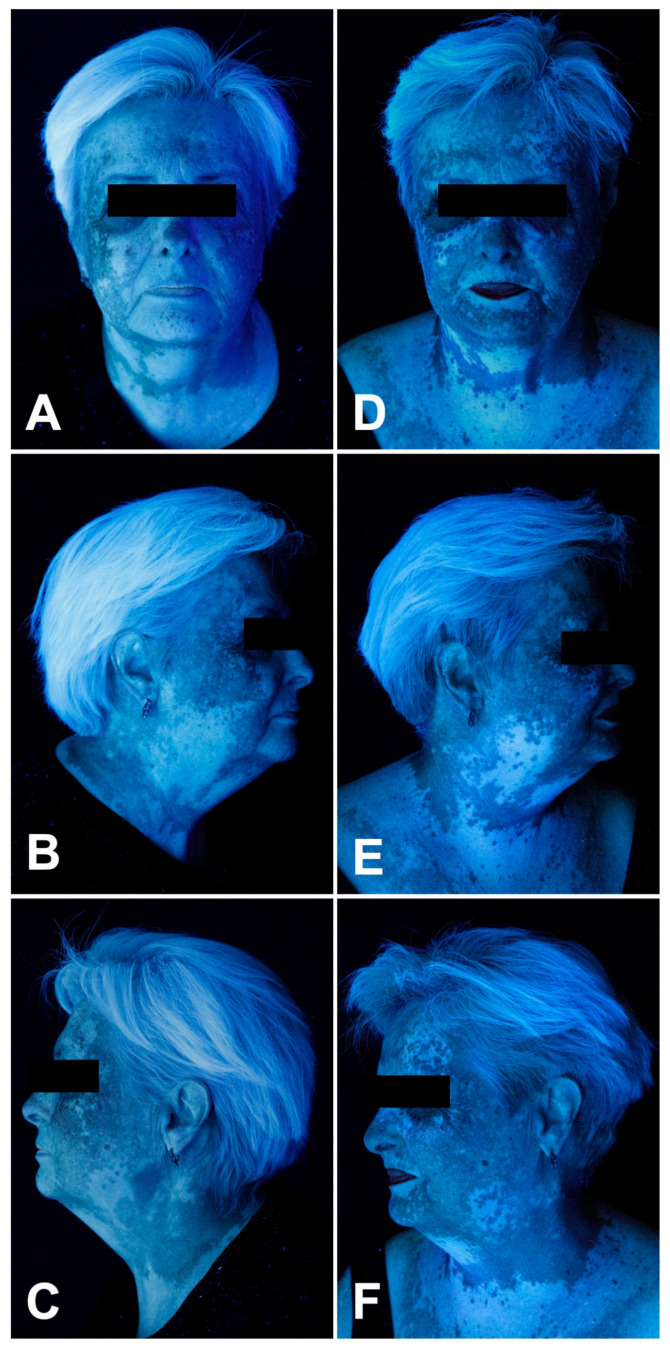
Patient 2: Clinical skin findings before (**A**–**C**) and after (**D**–**F**) six months of topical ruxolitinib 1.5% cream applied twice daily.

**Figure 4:**
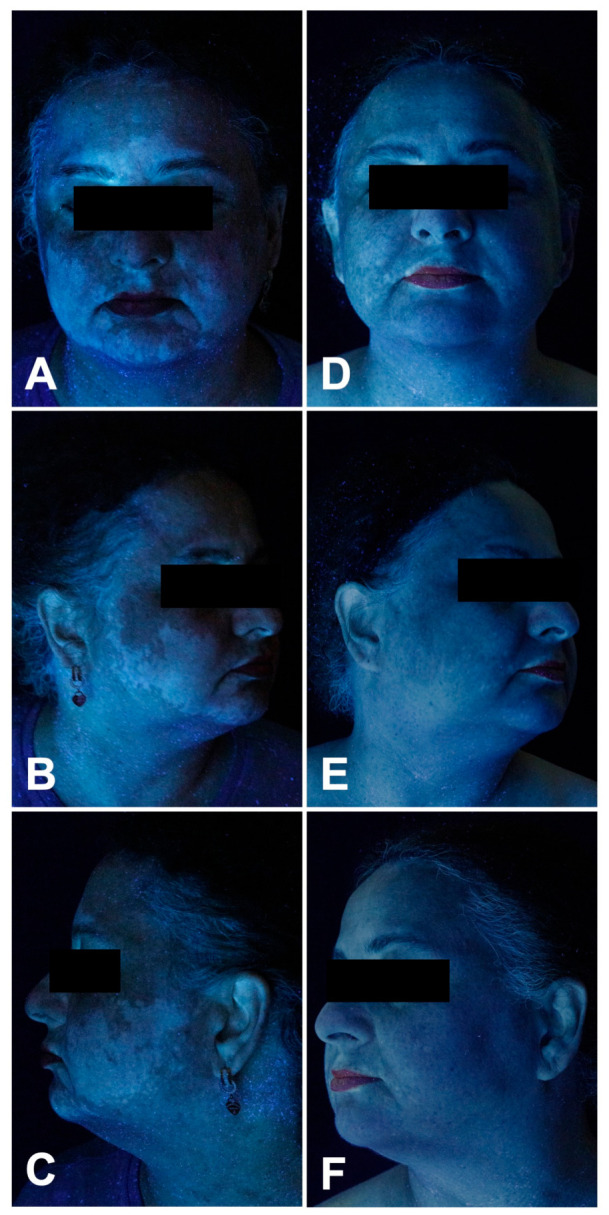
Patient 3: Clinical skin findings before (**A**–**C**) and after (**D**–**F**) twelve months of topical ruxolitinib 1.5% cream, applied twice daily.

The clinical skin findings were documented before and during treatment under Wood’s light (Figure 1, Figure 2, Figure 3 and Figure 4). This long-wave UV-A light (340 to 365 nm), causes the metabolites produced during the breakdown of melanocytes to fluoresce [23], making vitiligo more visible.

An estimate of the area of the face affected was made using the Facial Vitiligo Area Scoring Index (F-VASI) that is achieved by counting the number of imaginary thumbs (“fingertip units”) placed side by side (each corresponding to 0.1% of body surface area) [24].

To quantitatively assess the area affected by vitiligo patches, the software tool presented in [25] was further developed and adapted. The software features a semi-automatic detection of depigmented patches. An experienced operator, in this case a dermatologist, manually circled rough polygonal regions of interest (ROIs) containing the patches, and an image processing algorithm was then performed in each ROI to refine the pixels affected by vitiligo. The main steps of the detection algorithm can be summarised as:

(1) Image filtering: The three channels of the RGB images acquired under Wood’s light illumination are combined in different ways to obtain a black and white image that enhances the contrast between the patch and the healthy skin.
(2) Image binarisation: The obtained monochrome image is binarised with a specific threshold value, to obtain a 0–1 map (0 corresponds to healthy skin, while 1 corresponds to vitiligo patches).
(3) Area normalisation: The pixels identified as vitiligo patches are counted to quantify the image area affected by vitiligo. To ensure consistency between images with different size, resolution and face dimensions, the obtained area is normalised with respect to the total face area of the patient.
(4) Vitiligo index definition: Since multiple views of the same patient are available, to accurately represent the patches in different face regions, a unique parameter ranging from 0 to 1 for each patient is defined by combining the patches of each view.

A full description of this method was reported by Neri et al. [25].

With respect to the software presented in [25], some major developments are detailed below. Concerning Step 1, a new filter was implemented to enhance the image contrast. In particular, the contrast-limited adaptive histogram equalization (CLAHE) method [26] was adopted (implemented in the MATLAB (version R2025b) function adapthisteq [27]).

An example of the results obtained with this filter, compared with the filter used more often in [25], is shown in Figure 1. It shows the results obtained with the previous filter compared to the results of the newly implemented filter and illustrates how the CLAHE approach highlights the vitiligo patches compared to healthy skin. Even though this filter provides the best performance in the dataset, the operator may manually select a different filter. Additionally, the algorithm adaptivity to different skin regions was enhanced by developing a different binarisation procedure (Step 2). Instead of setting a global threshold value for all ROIs, the operator has the option of selecting a different threshold for each patch. Finally, a global vitiligo index for the patient was defined (Step 4). Since three different views are available for each patient (i.e., a frontal view, and the right and left side views) an index is preliminarily defined for each view by computing the ratio between the number of vitiligo pixels and the total face area of those views. Subsequently, the indexes obtained for each view are averaged to obtain the Face Vitiligo Index (FVI) following the equation:

$$FVI=\frac{1}{3}\sum_{i=1}^{3}\frac{p_{v,i}}{p_{f,i}}$$

According to this equation, for the i-th view (with i = 1, 2 or 3), *p_v,i_* represents the number of vitiligo pixels, while *p_f,i_* represents the total number of pixels of the face. If the FVI reaches 1, the patient’s skin is fully depigmented, while if the FVI approaches 0, the vitiligo patches are barely detectable. This parameter can be computed for each patient before and after treatment, to quantitatively assess the effect of therapy in inducing repigmentation.

## 3. Results

### 3.1. Patient 1

A 66-year-old female presented to our clinic with a swelling over the triceps brachii muscle of her right arm. Biopsy revealed a soft tissue metastasis of a sarcomatoid dedifferentiated amelanotic melanoma without a BRAF V600 mutation. Computed tomography (CT) and magnetic resonance imaging (MRI) staging examinations did not reveal any additional metastases.

**Figure 5:**
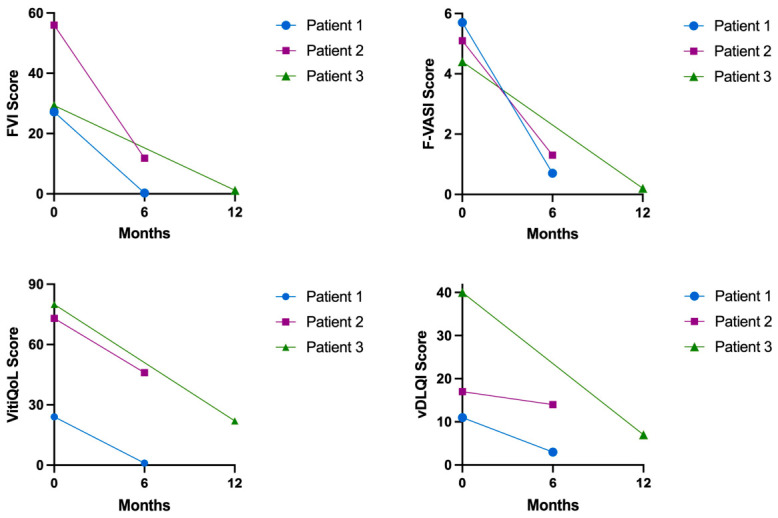
Face Vitiligo Index (FVI), Facial Vitiligo Area Scoring Index (F-VASI), vDLQI (Vitiligo Dermatology Life Quality Index), and Vitiligo-specific Quality of Life (VitiQoL) scores before and after treatment with 1.5% ruxolitinib cream; Patient 1: blue dot; Patient 2: purple square; Patient 3: green triangle.

The patient was diagnosed with stage IIIB N1c (American Joint Committee on Cancer 2017 (AJCC 2017)) melanoma of unknown primary [28]. The metastasis was resected and the patient received the anti-PD1 inhibitor pembrolizumab in an adjuvant setting. After the fifth dose, laboratory examinations showed ICI-induced thyroiditis CTCAE 2 (Common Terminology Criteria for Adverse Events [29]); hence levothyroxine was started. Following the eighth dose, the patient presented with progressive depigmentation affecting the face (F-VASI 5.7) (Figure 2A–C), chest, arms, and hands. Twice daily application of topical ruxolitinib 1.5% to the face and neck was initiated and pembrolizumab was continued. Upon follow-up six months later, a 95.7/97.7% repigmentation of the face (FVI 27.16 to 0.28; F-VASI 5.7 to 0.7) was observed (Figure 2D–F and Figure 5). The patient noted first effects after 10 weeks of application.

vDLQI reduced by 72.7% from 11 (max. 42) to 3 and VitiQoL by 95.8% from 24 (max. 90) to 1 (Figure 5). The patient developed neither local or systemic side effects, nor significant laboratory changes.

### 3.2. Patient 2

A 62-year-old female was referred to our clinic with a medical history of Stage IIB (AJCC 2017) [28] nodular melanoma on her right forearm, Breslow thickness 4.5 mm with lymphatic invasion.

Wide local excision with 2 cm safety margins and sentinel node biopsy revealed no further malignancy. The patient declined adjuvant therapy with anti-PD1 antibodies.

Six months later a satellite metastasis without BRAF V600 mutation was excised, hence stage IIIC (AJCC 2017) [28]. After another month, CT revealed pulmonary and splenic metastases, hence the stage shifted to IV M1c (AJCC 2017) [28].

The patient was started on combined immunotherapy with ipilimumab and nivolumab. She developed an ICI-induced rash CTCAE 1, self-limiting, after the first dose and experienced strong back pain during ipilimumab infusion. Treatment was thus switched to nivolumab monotherapy which was subsequently well tolerated. Immunotherapy was interrupted due to ICI-induced hepatitis CTCAE 3 that was treated with methylprednisolone. The patient also experienced ICI-induced adrenalitis CTCAE 2 that was well controlled with hydrocortisone treatment.

Subsequently, the patient developed progressive depigmentation of her face and neck (F-VASI 5.1) (Figure 3A–C). Topical ruxolitinib 1.5% cream was applied twice daily during ongoing nivolumab therapy. After six months, a 78.9%/74.5% (FVI 55.95 to 11.83; F-VASI 5.1 to 1.3) repigmentation was observed (Figure 3D–F and Figure 5).

The patient was very satisfied with the results, DLQI dropping from 17 (max. 42) to 14 (−17.6%) and VitiQoL dropping from 73 (max. 90) to 46 (−37.0%) (Figure 5).

No local or systemic side effects and no significant laboratory changes were observed.

### 3.3. Patient 3

A 46-year-old female patient presented to our clinic with external histopathology of an anal mucosal melanoma (pT4b). Colorectal surgeons performed a wide local excision with localisation-adapted safety margins in the anal canal and lower rectum. Histopathology showed multiple melanoma infiltrates. Sentinel node biopsy was not possible due to multiple tracer-positive lymph nodes in the bilateral inguinal regions, in the right iliac and left pararectal regions.

Four months later, the patient developed right inguinal lymph node metastases, multifocal hepatic metastases, as well as a local recurrence in the anal canal. Combined immunotherapy with ipilimumab and nivolumab was initiated. After two months, the patient developed ICI-induced colitis CTCAE 1, self-limiting and ICI-induced thyroiditis CTCAE 2, substituted with levothyroxine, as well as ICI-induced hepatitis CTCAE 3, managed with methylprednisolone.

After two months, fluorodeoxyglucose positron emission tomography–CT showed progressive disease with new lymph node metastases in the upper abdomen and the left hilum. Targeted therapy with encorafenib and binimetinib was initiated and well tolerated resulting in ongoing disease control for over three years.

During follow up the patient stated extensive, depigmentation affecting her face (F-VASI 4.4) (Figure 4A–C). It first appeared after two months of immunotherapy and slowly progressed, covering significant parts of the face. The patient was started on 1.5% ruxolitinib cream, applied to the face twice daily. After 12 months a 99.1/95.5% (FVI 29.32 to 1.16; F-VASI 4.4 to 0.2) almost complete repigmentation was observed (Figure 4D–F and Figure 5).

The patient was very satisfied with the repigmentation. DLQI dropped from 40 (max. 42) to 7 (−82.5%) and VitiQoL from 80 (max. 90) to 22 (−72.5%) (Figure 5).

Neither local or systemic side effects, nor significant laboratory changes were observed.

## 4. Discussion

Patients with vitiligo experience substantial psychosocial burden, frequently reporting feelings of stigmatisation, anxiety and depressive symptoms [30]. Quality-of-life impairment is particularly pronounced when the head and neck region are affected [31].

In patients with advanced melanoma, ICI-induced vitiligo has consistently been associated with favourable clinical outcomes [8,9,10,11,12,13,14]. However, for affected individuals, it can represent the only visible sign of disease or treatment, exposing them to uncomfortable social interactions and potentially implicit pressure to reveal their melanoma diagnosis. As a result, ICI-induced vitiligo should not be regarded as a purely cosmetic adverse event, but rather a condition with meaningful psychosocial consequences, the treatment of which can directly improve patient well-being. It should be acknowledged that melanoma-associated hypopigmentation (MAH) has been described in patients with melanoma independent of therapy and may phenotypically overlap with vitiligo [7]. However, in these patients, the consistent temporal association with ICI exposure and disease course make MAH an unlikely explanation, strongly supporting a diagnosis of ICI-induced vitiligo.

In this cohort of three patients with advanced melanoma and ICI-induced vitiligo affecting the face, topical ruxolitinib was associated with marked repigmentation effects and positive impact on patient-reported quality of life.

All three patients showed extensive repigmentation during follow-up (average 91.2/89.2% for FVI/F-VASI), accompanied by substantial improvements in vDLQI (mean reduction 57.6%) and VitiQoL (mean reduction 68.4%) without observed side effects.

Given the limited number of patients and the descriptive nature of this analysis, these observations should be interpreted as exploratory and hypothesis-generating rather than as evidence of clinical efficacy.

Topical ruxolitinib is only minimally absorbed systemically. In the phase III TRuE-V1 and V2 trials, steady state plasma concentration was measured at 26.4 and 28.4 nM, respectively, significantly below 281 nM, the half-maximum concentration associated with JAK-mediated myelosuppression in adult whole blood assays [17,32]. Even under maximum-use conditions, treating between 25 and 40% body surface area (BSA) in a study for patients with atopic dermatitis, no immunosuppression was detected, steady state plasma concentrations being reported at 104 nM [33].

As ruxolitinib was applied to only parts of the face (less than 4.5% of BSA [34]) in the present cohort, systemic adverse events were not expected. Importantly, when used in patients with melanoma undergoing treatment with ICI, no systemic immunosuppression that may affect intended immune responses against metastatic melanoma is anticipated. This is especially meaningful as long-term treatment with topical steroids, routinely used as first-line treatment in vitiligo, can cause permanent adverse effects including telangiectasia, acneiform eruptions, skin atrophy and striae, especially when applied to the face, chest and intertriginous areas. When applied to extensive skin areas over the prolonged periods frequently necessary to achieve and maintain repigmentation in vitiligo, systemic absorption has also been described as a concern [35].

From a clinical perspective, the primary value of the present work lies in drafting a feasible, low-threshold approach to the assessment and management of ICI-induced vitiligo rather than in guiding treatment decisions.

Topical ruxolitinib represents a non-invasive, locally acting option that may be particularly suitable for patients with facial involvement, in whom treatment burden, tolerability and cosmetic outcome are of high relevance.

The proposed combination of clinical photography, semi-automatic image analysis, and patient reported quality-of-life instruments could provide a framework for documenting disease burden and treatment response. While this approach requires consistent image acquisition and some technical expertise, it is reproducible in specialist dermatological settings and may facilitate more objective longitudinal assessment.

At the same time, several limitations must be acknowledged. The small sample size and absence of comparative data, as well as potential centre-specific factors, restrict generalisability and preclude conclusions regarding relative treatment effects. These considerations underscore the need for prospective analyses with appropriate control groups to better define its role alongside established therapeutic and diagnostic pathways.

## 5. Conclusions

To the best of our knowledge, this is the first quantitative analysis of the effects of a topical JAK inhibitor in ICI-induced vitiligo. Further studies are warranted to confirm these findings in broader patient cohorts, elaborate on this data and evaluate long-term efficacy as well as safety in a controlled study setting, including use beyond facial involvement.

## Data Availability

The original contributions presented in this study are included in the article. Further inquiries can be directed to the corresponding author.

## Abbreviations

The following abbreviations are used in this manuscript:

AJCC	American Joint Committee on Cancer
BSA	Body surface area
CLAHE	Contrast-limited adaptive histogram equalization
CT	Computed tomography
CTCAE	Common Terminology Criteria for Adverse Events
DLQI	Dermatology Life Quality Index
F-VASI	Facial Vitiligo Area Scoring Index
FVI	Face Vitiligo Index
ICI	Immune checkpoint inhibitor
irAE	Immune-related adverse event
JAK	Janus kinase
JAK1	Janus kinase 1
JAK2	Janus kinase 2
MAH	Melanoma-associated hypopigmentation
MRI	Magnetic resonance imaging
NSV	Non-segmental vitiligo
ROI	Region of interest
UV-A	Ultraviolet A
vDLQI	Vitiligo Dermatology Life Quality Index
VitiQoL	Vitiligo-specific Quality of Life
